# Encapsulated bacteria deform lipid vesicles into flagellated swimmers

**DOI:** 10.1073/pnas.2206096119

**Published:** 2022-08-15

**Authors:** Lucas Le Nagard, Aidan T. Brown, Angela Dawson, Vincent A. Martinez, Wilson C. K. Poon, Margarita Staykova

**Affiliations:** ^a^School of Physics and Astronomy, The University of Edinburgh, Edinburgh EH9 3FD, United Kingdom;; ^b^Department of Physics, Durham University, Durham DH1 3LE, United Kingdom

**Keywords:** active matter, motile bacteria, *Escherichia coli*, lipid vesicles

## Abstract

We study a synthetic system of motile *Escherichia coli* bacteria encapsulated inside giant lipid vesicles. Forces exerted by the bacteria on the inner side of the membrane are sufficient to extrude membrane tubes filled with one or several bacteria. We show that a physical coupling between the membrane tube and the flagella of the enclosed cells transforms the tube into an effective helical flagellum propelling the vesicle. We develop a simple theoretical model to estimate the propulsive force from the speed of the vesicles and demonstrate the good efficiency of this coupling mechanism. Together, these results point to design principles for conferring motility to synthetic cells.

## Significance

Swimming bacterial pathogens can penetrate and shape the membranes of their host cells. We study an artificial model system of this kind comprising *Escherichia coli* enclosed inside vesicles, which consist of nothing more than a spherical membrane bag. The bacteria push out membrane tubes, and the tubes propel the vesicles. This phenomenon is intriguing because motion cannot be generated by pushing the vesicles from within. We explain the motility of our artificial cell by a shape coupling between the flagella of each bacterium and the enclosing membrane tube. This constitutes a design principle for conferring motility to cell-sized vesicles and demonstrates the universality of lipid membranes as a building block in the development of new biohybrid systems.

The interaction of active and passive matter lies at the heart of biology. Active matter (1) consists of collections of entities that consume energy from their environment to generate mechanical forces, which often result in motion. Thus, the cell membrane, whose essential component is a passive lipid bilayer, can actively remodel during cell growth; motility; and, ultimately, cell evolution, under the forces exerted by a host of active agents. For example, continuous polymerization–depolymerization of actin in the cytoskeleton deforms the eukaryotic cell membrane into two- and one-dimensional protrusions (lamellipodia and filopodia) that can move the whole cell (2, 3). Similar actin-supported membrane protrusions are thought to have facilitated the accidental engulfment of bacteria that led to the emergence of eukaryotic cells (4). The biophysics of active membranes has therefore been subjected to interdisciplinary scrutiny (5). More recently, learning how to create active membranes systems that deform, divide, and propel has become a priority area in the drive to synthesize life ab initio (6).

Lipid vesicles enclosing natural or artificial microswimmers are becoming a model system for studying active membranes in vitro (7–15). Such composites have also direct biological relevance. For instance, from inside their eukaryotic hosts, bacterial pathogens such as *Rickettsia rickettsii* or *Listeria monocytogenes* (16, 17) continue their life cycles by hijacking the actin polymerization–depolymerization apparatus of their hosts and pushing out a tube-like protuberance from the plasma membrane. The pathogens then contact other host cells or escape into the surrounding medium by means of these membrane tubes (18).

To date, research in coupling swimmers with membranes has mostly been theoretical and numerical. Such models have predicted a range of interfacial morphological changes and, in some cases, net motion of the interface (7–13). The experimental realization of these systems was only recently achieved by encapsulating swimming *Bacillus subtilis* bacteria (14) and synthetic Janus particles (15) in giant lipid vesicles. Both experiments reported nonequilibrium membrane fluctuations and vesicle deformations, ranging from tubular protrusions to dendritic shapes. However, net motion of the vesicles was not observed in either case.

Here we present a similar experimental design but with markedly different outcome. *Escherichia coli*, another common motile bacterium, also extrudes membrane tubes but in addition sets the whole vesicle into motion. We demonstrate that such motion is due to a physical coupling between the flagella bundle of the enclosed cells and the tubes. The tube–flagella composite functions as a helical propeller for the entire vesicle.

In biology, the specificity of interactions between bacteria and the membranes of eukaryotic hosts underlies the plethora of parasitic and symbiotic relations that have emerged between cells (18–20). Likewise, our observations illustrate the importance of small details in the design of active matter systems (21). Encapsulated bacteria propelled by a single bundle of helical flagella can generate net motion of the vesicles, whereas encapsulated swimmers propelled at similar speeds by phoresis fail to do so (15). These observations illustrate the fact that it is dangerous to proceed from coarse-grained simulations or theory that neglect such details to predict the behavior of particular systems. At the same time, our results point to a design principle for conferring motility to artificial cell models.

## Results

### Encapsulated Bacteria Extrude Lipid Tubes.

We encapsulated a smooth-swimming strain of K-12–derived *E. coli* in giant unilamellar vesicles (GUVs) made of 1-palmitoyl-2-oleoyl-sn-glycero-3-phosphocholine lipids (POPC) and doped with a fluorescent dye using the inverted emulsion method (22). The internal medium was lysogeny broth (LB) supplemented with sucrose, which the bacteria cannot metabolize. The external medium was an aqueous solution of glucose, which, like sucrose, does not diffuse across the lipid membrane. Our GUVs sedimented toward the glass bottom of the sample chambers, which had been pretreated with bovine serum albumin to minimize vesicle adhesion. Bacteria and vesicles were imaged with an inverted microscope in phase-contrast and fluorescence modes. We occasionally observed cell division in the GUVs, and the bacteria remained motile for at least ∼8 h, thanks to nutrients provided in the inner medium and/or endogenous metabolism (23) enabled by the diffusion of dissolved O_2_ through the membrane.

**Fig. 1.**
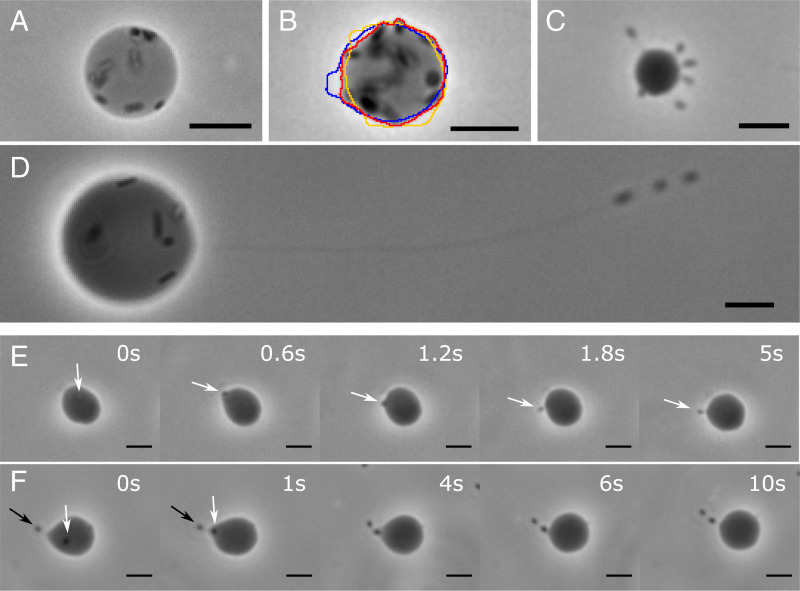
Bright-field images of GUVs in the course of osmotic deflation displaying membrane fluctuations and tubular protrusions. (*A*) Tense GUV encapsulating ∼10 bacteria (dark and lighter spots within the GUV) and displaying no visible fluctuations. (*B*) Deflated GUV displaying bacteria-amplified fluctuations illustrated by three superimposed contours. (*C*) GUV displaying six tubular protrusions extruded by bacteria, each containing a single cell. (*D*) GUV displaying a long (≳100 µm) tube with three cells at its extremity. (*E*) Image sequence showing a single cell (white arrow) extruding a tube by pushing on the membrane. (*F*) Image sequence recorded on a different GUV, showing a second cell (white arrow) entering a tube previously extruded by a single bacterium (black arrow), which leads to elongation of the tube. (Scale bars, 10 µm.)

In sample chambers sealed immediately after bacteria encapsulation, the majority of GUVs appeared tense and spherical, with no visible sign of shape fluctuations (Fig. 1*A*). The ∼10 encapsulated bacteria typically swam just below the inner surface, reminiscent of previous observations of *E. coli* in lecithin-stabilized spherical water-in-oil emulsion droplets (24). Among these GUVs, we occasionally observed GUVs with tubular protrusions containing one or more bacteria.

To increase the number of GUVs exhibiting such protrusions, we used osmotic deflation to decrease the membrane tension of our vesicles by keeping sample chambers open for a defined period of time, $t_{\text{w}}$, before sealing for observation. Water evaporation increased the osmolarity of the external solution by a controlled factor *α* (*Materials and Methods*), leading to water efflux. Progressive deflation yielded a higher fraction of GUVs with bacteria-containing tubes than sudden mixing with a hypertonic buffer. At $t_{\text{w}}=0$, the internal and external media were isotonic (*α* = 1), giving mostly tense vesicles with only a fraction χ=0.17 showing bacteria in tubular protrusions (see *SI Appendix* for details). At even a moderate deflation, α=1.05, some vesicles exhibited pronounced fluctuations at $t_{\text{w}}\approx 20\;\text{min}$ and χ=0.49. Further deflation, α=1.1, gave $\chi =0.67,\;t_{\text{w}}\approx 40\;\text{min}$, with tube-bearing vesicles dominating our field of view (Fig. 1*C*). The vesicles displayed either multiple tubes (Fig. 1*C*) or a single tube with one or multiple bacteria inside (Fig. 1*D*).

We were able to capture occasionally the rapid process of the initiation of tubular protuberances by swimming bacteria. This takes ∼1 s and requires a cell to swim perpendicular to the membrane (Fig. 1*E*). A second cell can enter an already formed tube (Fig. 1*F*), causing its extension. Tube elongation consumes membrane and generates tension (Fig. 1*F*), resulting in a more spherical shape and preventing further tube growth. Interestingly, a bacterium swimming into a preexisting cell-free membrane tube was never observed. Moreover, no bacteria-containing tubes were seen with encapsulated dead cells, and bacteria swimming outside vesicles do not pull off membrane tubes under our conditions (*SI Appendix*).

### Bacteria in Membrane Tubes Propel Lipid Vesicles.

**Fig. 2.**
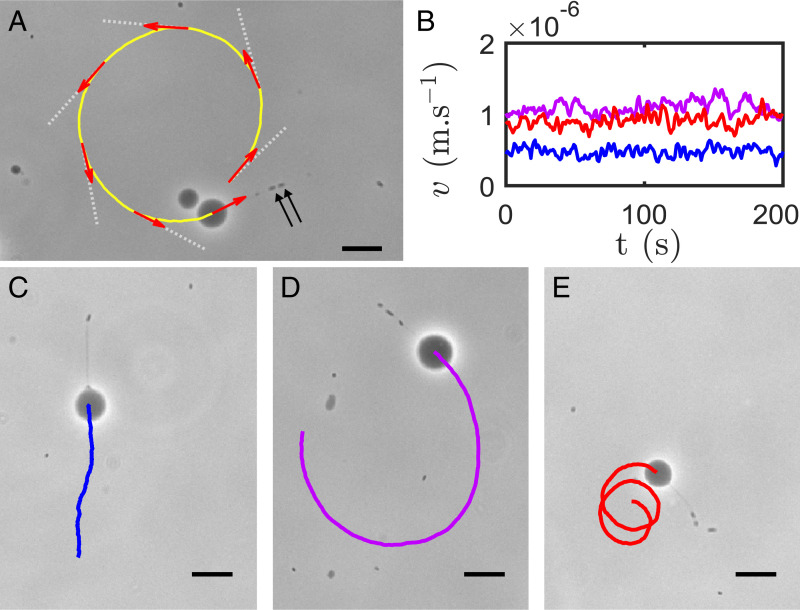
Propulsion of GUVs containing *E. coli* cells. (*A*) Trajectory (yellow line, Δt=180 s) of a GUV propelled by two bacteria in a membrane tube (black arrows). Red arrows indicate the orientation of the instantaneous velocity vector at 30-s intervals, and white dotted lines indicate the orientation of the tube at the same time points, showing that the GUV swims tube-first. (*B*) Speed as a function of time for the three GUVs displayed in *C*–*E*, with matching colors. (*C*–*E*) Vesicle trajectories recorded over 200 s may vary from (*C*) straight line, to (*D*) counterclockwise, to (*E*) clockwise rotation. (Scale bars, 20 µm.)

In striking contrast to GUVs encapsulating Janus particles or *B. subtilis* bacteria, which display large deformations but remain static (14, 15), we observed that motile *E. coli* in membrane tubes were able to propel GUVs at typical speeds $v∼1\;\mu \text{m}\;{\text{s}}^{-1}$ (Fig. 2 and Movie S1). The motion is always tube-first with velocity vector parallel to the tube (Fig. 2 *A* and *C*–*E*). GUVs with bacteria but no tubular protrusions remain static, suggesting that motile bacteria in the vesicle lumen do not contribute to vesicle propulsion.

We found that vesicle trajectories varied from straight to curved clockwise (CW) and counterclockwise (CCW) within the same sample (viewed from the fluid side) (Fig. 2 *C*–*E*). Out of all assessed GUV trajectories (65 in total), 34% were curved CW, 42% were curved CCW, 20% were straight, and 5% displayed a reversal of their curvature during the tracking (the latter usually triggered by some rearrangement of the bacteria in the tube). Vesicle trajectories therefore tend to be curved, with no strong preference between CW and CCW rotation. This contrasts with unencapsulated bacteria outside vesicles, for which only 7% of circular trajectories near the glass slide (10 out of 146) were CCW. Such behavior, governed by hydrodynamic interactions between the bacteria and the glass substrate, agrees with previous measurements (25) and is consistent with a no-slip boundary condition operating at the slide (26). It is clear that these strong hydrodynamic effects do not translate into biased rotation of our vesicle–bacteria system, which is unsurprising given the more complex and variable geometry in that case.

### A Physical Coupling between Flagella and Membrane Tubes Generates a Propulsive Force.

**Fig. 3.**
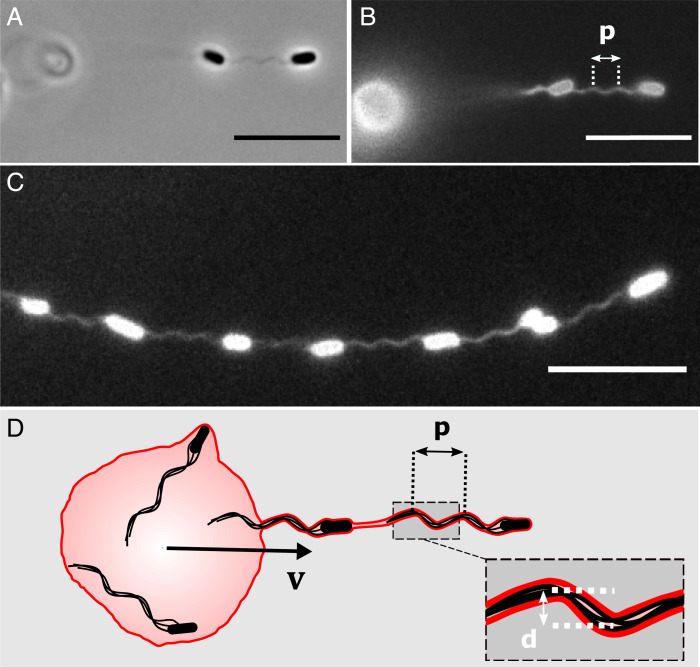
Membrane tubes propel GUVs by tightly coupling with the flagella bundles of enclosed cells. (*A*) Phase contrast image of two cells in a tube. The out-of-focus GUV is barely visible on the left. Close inspection reveals that the portion of tube connecting the two cells is helical. (*B*) Fluorescence image of the lipid tube displayed in *A*, confirming its helical shape with pitch p=2.3 μm. The fluorescence signal is emitted by the dye embedded in the GUV membrane. The bright disk on the left is the out-of-focus GUV. The tube appears blurred behind the second cell in the tube (i.e., closer to the GUV) in *A* and *B* due to the small depth of focus. (*C*) Fluorescence image of a tube containing multiple bacteria, showing the coupling with the flagella bundles behind each cell. (*D*) Schematic of the system (not to scale) describing the mechanism of GUV propulsion. The membrane (red contour) of the tube wraps the bacteria and adopts the shape of their helical flagella (pitch *p*, helical diameter *d*). Flagellar rotation within the tube generates a thrust force and results in an instantaneous velocity vector **v** parallel to the tube. (Scale bars, 10 µm.)

The self-propelled motion of our biohybrid vesicles is a surprising phenomenon because encapsulated bacteria are isolated from the external medium by a lipid membrane. To get insights into its mechanisms, we turn to high-resolution microscopy. Imaging the membrane directly by combining phase contrast microscopy with fluorescent imaging (Fig. 3) shows bacteria tightly wrapped in tubes with $R_{\text{t}}≲0.5\;\mu \text{m}$ along the whole length of any tube. The portion of tube behind each bacterial body is also noticeably thinner than the cell body itself. Knowing that the nearly rigid helical flagella bundle in swimming *E. coli* has a helical diameter *d* comparable to that of the cell body (27), this observation suggests that the portion of tube surrounding the flagella bundle is severely distorted from a cylindrical form.

Our images indeed show that the two-dimensional projection of the membrane around the flagella bundle has a sinusoidal shape (Fig. 3 *A*–*C*). We fitted the images of 10 helical tubes to a sine function (*SI Appendix*, Fig. S2) and determined the pitch p=2.3±0.2 μm and helical diameter d=0.4±0.1 μm (mean ± SD), which agree with previous values for the flagella bundle of *E. coli* (27). High-speed imaging returned rotation frequencies of typically 50 to 120 Hz (e.g., 90 Hz for the tube in Fig. 3*B*), which again falls within the range measured for the flagella bundle of *E. coli* (28) (Movie S2). We conclude that the lipid membrane wraps closely enough around the flagella bundle to adopt its helical shape and that the bundle retains the same geometry as in free-swimming cells.

These findings suggest a propulsion mechanism that is most easily applied to the simplest, single-tube vesicle (Fig. 3*D*). The thin membrane tube couples to the rotating helical flagella bundle of the nested bacterium, adopts its shape, and undergoes helical motion, which generates a thrust force. Consistent with this, we expect the propulsive force to be proportional to the number of bacteria in the tube if cell–cell interactions remain negligible.

### The Propulsive Force Scales with the Number of Bacteria in a Tube.

To verify this, we consider motile vesicles propelled by a single bacteria-bearing membrane tube and exclude cases such as that in Fig. 1*C*. The relevant Reynolds number is Re $=2Rv\rho_{\text{os}}/\eta ∼10^{-5}$, where R∼10 μm is the GUV radius, $\rho_{\text{os}}$ is the volume mass of the outer solution, and η∼1.5 mPa s is its viscosity at 20 ${}^{{}^{\circ}}$C (29, 30). Inertia is therefore negligible, and the propulsive force generated by the bacteria in a membrane tube, $F_{\text{prop}}$, is exactly balanced by the total drag force, $F_{\text{drag}}=\xi v$, acting on the composite swimmer (GUV + tube), where *ξ* is its friction coefficient and *v* is its speed:

[1] $$F_{\text{drag}}=\xi v=F_{\text{prop}}.$$

We include the contribution of the GUV and of the membrane-wrapped bacteria to *ξ* but neglect the much thinner, empty portions of tube (*SI Appendix* and *SI Appendix*, Fig. S5). We also neglect wall drag because we cannot quantify the GUV–wall distance. Thus, for a GUV bearing a single N-bacteria tube, $\xi =\xi_{v}+N\xi_{b}$, with *ξ_v_* and *ξ_b_* being the friction coefficients of the spherical GUV and of a single membrane-wrapped *E. coli*, respectively. A nonslip boundary condition applies to GUVs in flow (31), so that for a spherical GUV of radius *R*, $\xi_{v}=6\pi \eta R$. Modeling *E. coli* cells as oblate ellipsoids of minor and major axes *a* and *b* leads to $\xi_{b}=\frac{2\pi \eta b}{\ln\;\left(2b/a\right)-1/2}$ (23). We therefore have

[2] $$\xi =6\pi \eta R+\frac{2N\pi \eta b}{\ln\;\left(2b/a\right)-1/2}.$$

We take a=1 μm and b=3 μm throughout.

**Fig. 4.**
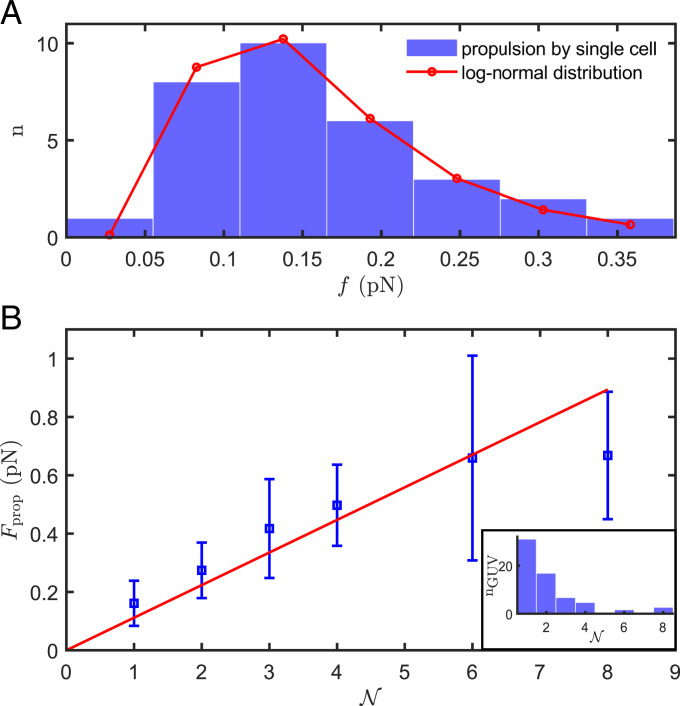
Propulsive force generated by bacteria in membrane tubes. (*A*) Histogram of *f*, the propulsive force estimated for all GUVs propelled by a single cell in a tube (*n* = 31). The distribution is described by a log-normal probability density function: $\text{P}(f)=\frac{1}{\sqrt{2\pi }f\sigma }\text{exp}\left(-\frac{{\left(\text{ln}(f)-\mu \right)}^{2}}{2\sigma^{2}}\right)$ with parameters μ=−1.9 and σ=0.48 for *f* in pN (converted to discrete values; red line), leading to a mean value $\bar{f}=0.16$ pN. (*B*) Average value of the total propulsive force $F_{\text{prop}}$ generated by bacteria as a function of the number of bacteria N in the tubes. The red line is a linear fit weighted by the inverse of the variance of the data, with a fitted slope 〈f〉=0.11N pN ($R^{2}=0.8$). Error bars correspond to SDs. (*Inset*) Number $n_{\text{GUV}}$ of GUVs tracked for each value of N.

Consider first only vesicles propelled by a single cell in a tube, for which we define $F_{\text{prop}}(N=1)=f$. Applying Eq. **1** using measured vesicle speeds and calculated *ξ* as inputs, we find a range of *f* values (Fig. 4*A*). Unlike *v*, we expect the propulsive force to be independent of the vesicle radius, and indeed we find no correlation between *f* and *R* (*SI Appendix*, Fig. S3). Instead, the observed variability in *f* is likely due to the distribution of single-bacterium swimming speeds [as revealed by differential dynamic microscopy (*SI Appendix*, Fig. S4) (32)] and variable (but unknown) vesicle–wall hydrodynamic interactions. Variations in tube radius around the flagella bundle are predicted to contribute only weakly to this variability (*SI Appendix* and *SI Appendix*, Fig. S6). The spread in *f* is well described by a log-normal distribution with a mean of 0.16 pN, about three times lower than the thrust generated by a free-swimming *E. coli* (28, 33). However, since we neglect wall drag (34), our estimate of *f* is a lower bound. The true mean *f* may therefore be statistically indistinguishable from the propulsive force of a free-swimming cell, revealing a surprisingly efficient coupling between the encapsulated flagella with the external fluid via the surrounding lipid membrane.

Turning to multibacteria tubes, we used Eqs. **1** and **2** and deduced $F_{\text{prop}}$ for GUVs propelled by tubes containing up to eight bacteria (Fig. 4*B*). There is significant variability in the deduced values, especially at large N. The sources of variability previously mentioned for the distribution in single-cell *f* values continue to be relevant here, with potentially the addition of variable cell–cell interactions. Moreover, only a small number of GUVs were tracked for N≥6 ($n_{\text{GUV}}\leq 3$ in each case) because larger GUVs with longer tubes lead to a higher probability of them interacting with other GUVs, precluding their use for data collection.

With these caveats, we find $F_{\text{prop}}=N\langle f\rangle$, with a fitted 〈f〉=0.11 pN (regression coefficient = 0.8 with the origin included as a data point), consistent with the single-cell force estimate of 0.16 pN. Such proportionality confirms that individual bacteria in a membrane tube are independent sources of propulsion, with negligible hydrodynamic and other interaction between them.

## Discussion

**Fig. 5.**
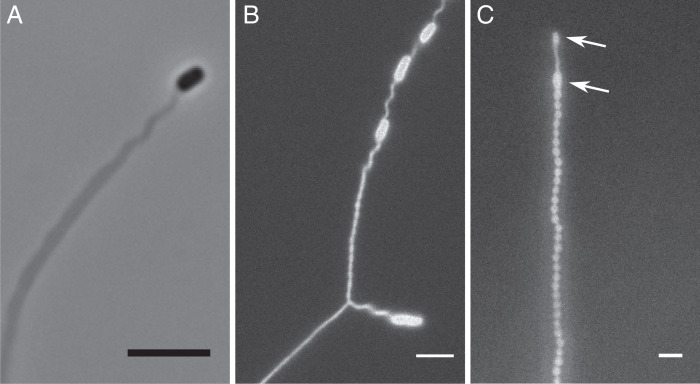
Thicker, split, and pearled tubes. (*A*) Thicker tube coupling with the flagella bundle of an enclosed cell. (*B*) Tube split into two subtubes, one containing a single cell and the other one containing four bacteria (only three are visible). (*C*) Pearled tube with two bacteria at its end (white arrows). (Scale bars, 5 µm.)

The tube size appears to be an important factor for the propulsion of vesicles. GUVs with very short tubes will not move because flagella bundles cannot contribute to the propulsion from the vesicle lumen (Fig. 3*D*). On the other hand, only tubes that are sufficiently thin will couple to the shape of the rotating flagella and undergo a helical motion. Indeed, the majority of our observations depict tubes closely wrapped around the flagella bundles, but in some rare cases we also observed thicker thrust-generating tubes (diameter ∼1 μm) (Fig. 5*A* and Movie S3). This implies that a tight, no-slip coupling of flagella bundle and membrane is not essential to the propulsion; flagella can generate a propulsive deformation of the membrane also through an interstitial aqueous layer. Our considerations are supported by theoretical studies predicting the same propulsion speeds for flagella undergoing a rigid helical rotation and a helical wave deformation (35). In either case, as long as the membrane tube is thin enough to be perturbed by the enclosed rotating bundle, it adopts its helical shape and functions as an effective flagellum generating propulsion for the whole vesicle.

Interestingly, however, the thin tubes in our experiments do not agree with the classical elastic theory of membrane tubulation (36), which predicts tube radii $R_{t}=\frac{2\pi \kappa }{F}∼1.3\;\mu \text{m}$, where $\kappa ∼10^{-19}\;\text{J}$ is the bending rigidity of a POPC membrane (37), and F≈0.5 pN is the thrust force generated by a free-swimming *E. coli* (28, 33).

Such discrepancies are usually reconciled by the existence of a positive spontaneous membrane curvature (38) and/or a relative excess of lipids in the outer membrane leaflet (39, 40). Both favor thin membrane protrusions (41) and are consistent with our observations of spontaneously occurring thin tubes after deflation, even in bacteria-free GUVs. Control experiments suggest that a transmembrane lipid density asymmetry is a more likely mechanism (*SI Appendix* and *SI Appendix*, Fig. S7). The inverted emulsion encapsulation protocol offers a much longer time for outer leaflet equilibration than for the inner leaflet, which, combined with the slow adsorption of POPC lipids to oil/water interfaces (≳1 h to reach saturation) (22), leads us to expect a difference in the lipid densities between the two leaflets. Vutukuri et al. used a similar method to produce GUVs and observed that their self-propelled Janus particles were able to extrude membrane tubes at much lower propulsive forces than the theoretically predicted tubulation force (15). Although likely to be of general interest, a detailed investigation of this issue and the physics associated with GUV production by the inverted emulsion method are beyond our scope.

For completeness, we also report here some rarer tube morphologies, which illustrate the richness of behaviors spontaneously occurring in our system. Bacteria can sometimes generate split tubes (Fig. 5*B*) with dynamic junctions moving along the tube (Movie S4). Pearling instabilities can also be observed, especially in thicker tubes, behind the section wrapping the flagella bundles (Fig. 5 *B* and *C*). Occasionally, cells divide in the tube, sometimes leading to interesting tube dynamics (Movie S5).

## Conclusion

We showed that motile *E. coli* swimming inside deflated GUVs can deform the lipid membrane and extrude tubular membrane protrusions, in agreement with recent studies using *B. subtilis* and synthetic Janus particles (14, 15). Whereas previous systems remained static, our vesicles display self-propelled motility. Detailed microscopy revealed that the membrane tube wraps the enclosed bacteria and follows the motion of their helical flagella bundle. Such a tube acts as an effective flagellum for the vesicle and provides the propulsive force needed to generate a sustained motion. In passing, we note that some bacteria, such as various gram-negative *Vibrio* species, have evolved to produce a membranous sheath around their flagella, the role of which remains poorly understood (42). Future biophysical studies exploring how membrane properties such as fluidity, stiffness, and phase separation affect the propulsion mechanism should therefore prove interesting.

The propulsion mechanism described here appears specific to flagellated bacteria coupled to membrane tubes. It clearly cannot operate for spherical Janus swimmers propelled by phoresis (43). The reason for the absence of vesicle motion with swimming *B. subtilis* is less clear (14). A run-and-tumble strain was used in that work, and propulsion in *B. subtilis* is generated by multiple flagella bundles (44). Furthermore, the vesicles were produced by electroformation rather than by the inverted emulsion method. Any of these differences might preclude the emergence of vesicle motility (or, indeed, the facile formation of stable membrane tubes). This highlights the specificity of active matter: there is no generic behavior of swimmers encapsulated in vesicles. Even two bacterial species swimming using flagella bundles produce different effects.

Simple biohybrid systems made of living bacteria encapsulated in synthetic vesicles have been explored for their biotechnological potential in shielding and delivering probiotic bacteria and for biosensing (45–48). Our work adds the possibility of these delivery vehicles becoming self-propelled, which might enable more targeted delivery. This could be achieved by exploiting bacterial chemotaxis or by using magnetotactic bacteria guided by magnetic fields (49).

Finally, we note that in the field of synthetic life, there has been progress in mimicking biological motility by the bottom-up generation of self-propulsion using entirely synthetic components (50). At the same time, novel biohybrid systems have been generated by coupling synthetic lipid membranes to living cells or isolated cell components (51). Using the latter approach, it has long been possible to tow lipid vesicles by externally applied forces, i.e. using actin polymerization–depolymerization (52) or by attaching bacteria to vesicles (53). The present work reports synthetic vesicle propulsion by *internally* generated forces, in our case by the encapsulation of living cells.

## Materials and Methods

All data and analysis codes can be found in the following data repository: https://doi.org/10.15128/r2rn3011433.

### Bacterial Cultures.

We used strain AD83, a smooth-swimming strain derivative of *E. coli* AB1157. This strain was constructed by deleting the *cheY* gene (23) and further transformation with plasmid pWR21 (54), which expresses eGFP constitutively and confers resistance to Ampicillin. Ampicillin was added to all solutions at a final concentration of 100 µg mL^−1^.

Bacteria were plated on lysogeny broth (LB, Miller’s formulation) agar plates and grown at 37 ${}^{{}^{\circ}}$C to form isolated colonies. LB supplemented with 400 mM sucrose (LB-sucrose), filter-sterilized using a 0.2-µm filter after sucrose addition, was used thereafter. Unless otherwise specified, mM refers to solute concentrations expressed in millimoles of solute per liter of solvent.

Single colonies were grown overnight in 5 mL of LB-sucrose in an orbital shaker incubator (37 ${}^{{}^{\circ}}$C, 200 rpm). Then 10 mL of fresh LB-sucrose was inoculated with 100 µL of overnight culture and incubated aerobically for 3.5 h to an optical density OD∼0.7. Harvested cells were centrifuged (6,500 × *g*, 2 min), redispersed in fresh LB-sucrose, and diluted to OD=0.3 with fresh LB-sucrose.

### Lipids/Oil Solution.

POPC dissolved in chloroform was purchased from Avanti Polar Lipids. All other chemicals were purchased from Sigma-Aldrich. The dye 1,1${}^{\prime }$-dioctadecyl-3,3,3${}^{\prime }$,3${}^{\prime }$-tetramethylindocarbocyanine perchlorate (DiIC_18_) was stored in ethanol (0.58 mM stock solution) and kept in the dark at $4{\;}^{{}^{\circ}}$C. Lipids/oil solutions were prepared as follows. Thin films of POPC and DiIC_18_ were deposited in separate vials by evaporating the solvents of the respective stock solutions (chloroform and ethanol, respectively) under gentle nitrogen (N_2_) flow and dried in vacuum for 2 h. Light mineral oil was added to the POPC vial to a final POPC concentration of 2 mg mL^– 1^, and the lipids were dispersed by sonicating for 1.5 h. The resulting POPC/oil solution was transferred in the vial containing DiIC_18_ and sonicated for a further 30 min in cold water to disperse the dye (99.5:0.5 POPC:DiIC_18_ molar ratio). Lipids/oil solutions were stored for up to a week at room temperature in N_2_ and sonicated for ∼10 min before experiments.

### Bacterial Encapsulation in GUVs.

Bacteria were encapsulated in GUVs using the inverted-emulsion method (22). Briefly, a lipid monolayer was formed at an oil/water interface by layering 200 µL of lipids/oil solution on top of 800 µL of aqueous buffer in a 1.5-mL microcentrifuge tube, and the tube was incubated at room temperature for 1 h. The aqueous buffer (deionized water supplemented with 840 mM glucose, filter-sterilized) is the outer solution (OS) surrounding the GUVs at the end of the protocol. Protocol steps were timed to ensure that the bacterial suspension was ready shortly before the end of the incubation period.

In a separate tube, 10 µL of bacterial suspension was emulsified in 200 µL of oil/lipids solution by dragging the tube ∼15 times on a tube rack, allowing the formation of lipid-coated water-in-oil droplets. The emulsion was quickly layered on top of the column prepared as described above, and the tube was centrifuged at 1,500 × *g* for 5 min to transfer the droplets through the preformed lipid monolayer, thus forming GUVs. Droplet transfer was aided by a small density difference between the OS and the LB-sucrose inner solution (IS) ($\rho_{is}=1.0593$ g cm^– 3^, $\rho_{os}=1.0512$ g cm^– 3^). After carefully removing the oil from the tube, GUVs were collected using a micropipette and redispersed in another tube containing fresh OS. That tube was centrifuged at 1,000 × g for 1 min and the supernatant discarded to remove most oil droplets and lipid aggregates from the suspension. The final suspension, used in experiments, was obtained by redispersing the washed GUVs into fresh OS.

### Imaging and Analysis.

Sample chambers (∼1×1×0.1 cm^3^) were prepared by bonding polydimethylsiloxane (PDMS) spacers to glass coverslips using a plasma cleaner (Zepto, Diener electronic). Just prior to use, chambers were treated for 10 min with a freshly prepared solution of bovine serum albumin (BSA) dissolved in OS at 10% wt/vol to minimize GUV adhesion to the glass and washed thoroughly three times with OS.

Open chambers were filled with 190 µL of GUV suspension. Using a volume of liquid greater than the chambers’ volume helped avoid trapping air bubbles while sealing the chambers after evaporation. We used an evaporation-based deflation protocol to obtain softer GUVs. A sample of initial mass *m_i_* with 840 mM glucose (molar mass *M_g_*) in deionized water OS is evaporated to final mass *m_f_*:

[3] $$m_{f}=\frac{m_{i}}{\alpha }\frac{1+\alpha c_{0}M_{g}}{1+c_{0}M_{g}},$$

where $c_{0}=0.84$ mol kg^– 1^ is the initial solute osmolality.

GUVs were imaged near the BSA-treated bottom coverslips using a Nikon TE2000 inverted microscope with Nikon phase contrast objectives (Plan Fluor 20×/0.5 Ph1 and Plan Apo 60×λ/1.4 Ph3 Oil). Movies were recorded using an scientific Complementary Metal–Oxide–Semiconductor camera (Orca Flash 4.0, Hamamatsu) and Micromanager (55) typically for 3 to 5 s at 10 frames per second (fps). High-magnification movies were recorded at 400 fps for 2 to 5 s to study the motion of helical tubes. We imaged at 1 fps for 250 s to study GUV motion, tracking them in MATLAB R2020a using a code adapted from ref. 56 and only analyzing vesicles showing >30 s tracks. Instantaneous GUV speeds come from linear fitting of the trajectories over four consecutive frames (Δt=3 s between frames). Whenever possible (∼80% of tracked vesicles), we corrected for advection due to occasional weak residual flows after sealing the sample chambers by tracking a bacteria-free vesicle in the vicinity of motile GUVs and subtracted its drift velocity. The rest of the image analysis was performed using Fiji (57).

### Differential Dynamic Microscopy.

Bacteria grown in LB-sucrose were centrifuged (6,500 × *g*, 2 min), diluted to OD=0.1 with fresh LB-sucrose, and used immediately to fill rectangle glass capillaries (VitroCom, inner diameters 0.40 mm × 8 mm) subsequently sealed with petroleum jelly (Vaseline) to prevent evaporation. Bacteria were imaged with a phase contrast objective (Plan Fluor 10×/0.3) ≈150 μm above the bottom of the capillaries. The 40-s movies were recorded at 100 fps for analysis (32).

## Supplementary Material

Supplementary File

Supplementary File

Supplementary File

Supplementary File

Supplementary File

Supplementary File

## Data Availability

Data have been deposited in Durham University (DOI: 10.15128/r2rn3011433) (58).
