# Supplementary material for: Encapsulated bacteria deform lipid vesicles into flagellated swimmers
Source: Proc Natl Acad Sci U S A. 2022 Aug 15;119(34):e2206096119. doi: 10.1073/pnas.2206096119 (PMC9407364; doi:10.1073/pnas.2206096119)
Supplement: Supplementary File [file pnas.2206096119.sapp.pdf]

PNAS
www.pnas.org

Supplementary Information for

Encapsulated bacteria deform lipid vesicles into flagellated swimmers

Lucas Le Nagard, Aidan T. Brown, Angela Dawson, Vincent A. Martinez, Wilson C. K. Poon, Margarita Staykova

Wilson C. K Poon.
E-mail: w.poon@ed.ac.uk
Margarita Staykova.
E-mail: margarita.staykova@durham.ac.uk

This PDF file includes:

Supplementary text
Figs. S1 to S7 (not allowed for Brief Reports)
Table S1 (not allowed for Brief Reports)
Legends for Movies S1 to S5
SI References

Other supplementary materials for this manuscript include the following:

Movies S1 to S5

Supporting Information Text

1. Tubulation analysis

Statistics. Evaporation-induced deflation of GUVs leads to a variety of GUV morphologies, with a large variability in the number and length of tubular protrusions observed as well as in the number of bacteria present in these protrusions. The fraction of GUVs χ that display at least one cell in a tube after deflating the GUVs by a factor α quantifies how easily encapsulated bacteria can extrude a membrane tube and/or enter a spontaneously formed tube. To ensure that any observed difference was not simply linked to aging of the sample, we imaged the $\alpha = 1.1$ chamber before the $\alpha = 1.05$ one. The results of this analysis are presented in Table S1, which also includes additional information about the radii of the GUVs. GUVs that did not contain bacteria were not included. As expected for GUVs produced by the inverted emulsion method, a wide distribution of radii is obtained. To compare similar GUVs only, we repeated the analysis on a subpopulation of GUVs with radii varying between 5 µm and 10 µm, which represents $\sim 45\%$ of the GUV population in each chamber. This analysis yielded almost identical results: $\chi = 13\%$ ($\alpha = 1$), $\chi = 48\%$ ($\alpha = 1.05$) and $\chi = 68\%$ ($\alpha = 1.1$), confirming that deflating the GUVs promotes membrane tubes.

For each GUV included in the analysis of Table S1, we also estimated the concentration c of encapsulated bacteria by counting the cells present in the vesicle. Despite the relatively large uncertainty associated with this counting method – especially for high bacterial concentrations – we observed that χ did not seem to depend on c for the range of concentrations used in our experiments. The number averaged concentrations (sum of all measured concentrations divided by the number of analysed GUVs) were $c = (2.2 \pm 2.4) \times 10^9$ cells mL^{-1}, $c = (5.6 \pm 8.7) \times 10^9$ cells mL^{-1} and $c = (3.7 \pm 5.7) \times 10^9$ cells mL^{-1} (mean $\pm$ standard deviation) for $\alpha = 1$, $\alpha = 1.05$ and $\alpha = 1.1$ respectively. The cell concentration distributions are not symmetric, Fig. S1, so that the number averaged concentrations tend to be shifted towards higher values due to the presence of relatively rare GUVs that have a very high concentration of bacteria. We thus also report median concentrations: $c_\mathrm{m} = 1.4 \times 10^9$ cells mL^{-1}, $c_\mathrm{m} = 2.6 \times 10^9$ cells mL^{-1} and $c_\mathrm{m} = 1.4 \times 10^9$ cells mL^{-1} for $\alpha = 1$, $\alpha = 1.05$ and $\alpha = 1.1$ respectively. For a GUV with $R = 10$ µm, these values correspond to a median of 6 to 11 encapsulated bacteria.

Tube formation and GUV propulsion require motile encapsulated cells. A recent study using a pathogenic strain of *E. coli* showed that bacteria can adhere to the lipid membrane of GUVs (1). We exposed empty GUVs to an outer medium with motile bacteria (OD = 0.3), and saw no structures resembling bacteria-containing tubes. Also, no GUV motion was observed for bacteria-free GUVs exposed to non-encapsulated bacteria.

We also sought to confirm that only *motile* bacteria could extrude membrane tubes or enter spontaneously formed membrane protrusions. We encapsulated dead *E. coli* cells, obtained by heating a culture at 60 °C for 15 min followed by the evaporation protocol. No bacteria were observed in membrane tubes in these conditions. Even if empty membrane tubes sometimes form spontaneously during the evaporation protocol, active swimming is required for bacteria to be observed in membrane tubes.

2. Absence of correlation between f and R

Fig. S3 shows that the propulsive force exerted by a single cell f (deduced from measurements as detailed in the main text) is uncorrelated with the GUV radius R (Pearson correlation coefficient $r = 0.13$).

3. Differential dynamic microscopy analysis of freely-swimming bacteria in LB-sucrose

Differential dynamic microscopy (DDM) can characterise the speed distribution of motile *E. coli* by assuming that it takes Schulz form (2):

$$P(v) = \frac{v^Z}{Z!}\left(\frac{Z+1}{\bar{v}}\right)^{Z+1} e^{-\frac{v}{\bar{v}}(Z+1)}, \quad [1]$$

where $\bar{v}$ is the mean of the speed distribution and Z is related to the variance σ_v^2 of $P(v)$ via $\sigma_v^2 = \frac{\bar{v}^2}{Z+1}$. We used DDM to characterize an aerobic suspension of freely-swimming *E. coli* in LB-sucrose and measured $\bar{v} = 15.9 \pm 1.0\,\mathrm{\mu m\,s^{-1}}$ and $\sigma_v = 6.2 \pm 0.9\,\mathrm{\mu m\,s^{-1}}$, Fig. S4 (a). Uncertainties come from the fits of the DDM analysis. We then estimate the thrust force distribution for freely-swimming cells by multiplying the speeds of Fig. S4 (a) by $\frac{k_B T}{D}$, where D is the isotropic diffusion coefficient of an *E. coli* cell. For non-motile *E. coli* in motility buffer, $D_0 \sim 0.3\,\mathrm{\mu m^2\,s^{-1}}$ (3). We use $D = \frac{2}{3}D_0$ to account for the increased viscosity from sucrose addition. The force distribution of freely-swimming cells is shifted towards higher values compared to the one obtained from bacteria in membrane tubes, as expected, see main text Fig. S4 (b). However, both distributions have similar shapes and coefficients of variation ($c_v = 0.51$ for bacteria in membrane tubes compared to $c_v = 0.39$ for freely-swimming cells), confirming that biological variability contributes significantly to the spread in f observed in Fig. 4 (a) of the main text.

4. Drag on empty portions of tube

Our model presented in the main text takes into account the drag on the vesicle, ξ_v, and that of each bacterium in the tube, ξ_b, but it neglects the drag contribution from the empty portion of the tube, ξ_t, for the reasons stated below.

The drag coefficient of a cylindrical tube of length L_t and diameter D_t moving along its long axis is $\xi_t = \frac{2\pi\eta L_t}{\ln(2L_t/D_t) - 0.807}$ (4). Averaging over all tracked vesicles with experimentally measured tube lengths (subtracting a length of $3\,\mathrm{\mu m}$ per cell body) and vesicle radii, we find that the tube drag is small compared to that of the spherical vesicle, i.e. $0.19 < \frac{\xi_t}{\xi_v} = \frac{\xi_t}{6\pi\eta R} < 0.28$ for $0.05\,\mathrm{\mu m} < D_t < 0.4\,\mathrm{\mu m}$. (Note that the actual tube radii are below our optical resolution. Therefore we are exploring a range of plausible values). More importantly, we find no dependency of ξ_t on the number of bacteria present in the tube (the average $\frac{\xi_t}{\xi_v}$ for $D_t = 0.4\,\mathrm{\mu m}$ varies between 0.25 and 0.31 for all $\mathcal{N}$).

For comparison, the drag coefficient of a single bacterial cell is also relatively small; averaged over all GUVs it is $\frac{\xi_b}{\xi_v} = 0.10$. However, we must include the drag on the bacteria to avoid creating an $\mathcal{N}$-dependent error when plotting the force as a function of $\mathcal{N}$ in Fig. 4. This is not the case for ξ_t, the omission of which results in a slight underestimation of the calculated force but does not affect the linear trend between the propulsive force and $\mathcal{N}$. This is demonstrated in Fig. S5, which compares the forces calculated with and without taking ξ_t into account for $D_t = 0.4\,\mathrm{\mu m}$. The fitted force $\langle f \rangle = 0.13\,\mathrm{pN}$ (regression coefficient = 0.7) is similar to the value obtained in Fig. 4(b) without tube drag.

5. Variations in helix thickness weakly affect the propulsive force

Tube-to-tube variations in the thickness $2a$ of the helical portions of tube may affect the magnitude of the propulsive force and may contribute to the variability in f. Here, we show that this contribution is small over the plausible range of a in our experiments, $0.05\,\mathrm{\mu m} < 2a < 0.4\,\mathrm{\mu m}$.

We estimate the effect of a varying thickness on the generated thrust force using resistive force theory (RFT) (5, 6). We use Lighthill's drag coefficients C_n and C_t to estimate the thrust force generated by the rotation of a helix with pitch $p = 2.3\,\mathrm{\mu m}$, length $l = 5\,\mathrm{\mu m}$, helical diameter $d = 0.4\,\mathrm{\mu m}$ and rotation speed $\omega = 2\pi f_\Omega$ with $f_\Omega = 100\,\mathrm{Hz}$:

$$F_{\mathrm{RFT}} = \frac{\sin\phi}{2}\omega l d\,(C_n - C_t)\,, \qquad [2]$$

with $C_n = \frac{4\pi\eta}{\ln\frac{cp}{a\cos\phi} + 1/2}$, $C_t = \frac{2\pi\eta}{\ln\frac{cp}{a\cos\phi}}$, $c = 0.18$ and pitch angle ϕ given by $\tan\phi = \pi d/p$. As shown in Fig. S6, F_{RFT} varies weakly with a over the considered range. Note that RFT has been shown to significantly overestimate the thrust force (6), which explains why the force predicted here is larger than the thrust force of free-swimming *E. coli*. This still allows us to probe the dependency of the force on a and to show that we expect it to be negligible compared to other sources of variability, such as the biological variability demonstrated by DDM.

6. Tube formation in different experimental conditions

In our single-lipid GUVs, spontaneous curvature could arise from salt/sugar compositional asymmetry across the membrane (7, 8). To assess the reproducibility of the formation of active membrane tubes, we tested whether tube formation depends on the presence of specific molecules or on asymmetric conditions potentially leading to spontaneous curvature. We first verified that tube formation is unaffected in the absence of dye in the membrane, then tested the experimental conditions detailed below.

Symmetric, growth medium based conditions. We tested whether asymmetric sugar and salt concentrations are required for the formation of thin tubes. We encapsulated motile bacteria in GUVs using the protocol detailed in the main text, and diluted the GUV suspension 50 times in LB supplemented with 400 mM sucrose. After dilution, the outer medium thus consisted of slightly diluted LB supplemented with 392 mM sucrose and 17 mM glucose, very similar to the inner solution. The previously described evaporation protocol gave (sealed) samples at $\alpha = 1.1$. These conditions gave tubes similar to those observed in the main experiments, with many tubes being visibly thinner than the theoretical estimate of $R_{t,min} \sim 1.3\,\mu m$ given in the main text, (Fig. S7 (a)-(c)). This indicates that salt and sugar concentration asymmetry across the membrane is unlikely to be the main driver for tube formation. Taking advantage of the low density difference between the GUVs and the outer medium in these conditions, we also confirmed that bacteria-containing tubes can be seen while the GUVs are far from the BSA-coated substrate.

Defined buffers with sugar asymmetry. Using growth medium for the inner solution provides nutrients to the bacteria and minimizes the need to wash and transfer them into different buffers before encapsulation. However, LB is an undefined medium containing amino acids and peptides. We thus tested if these potentially membrane-active molecules are required for the formation of thin tubes by repeating the experiments with deionized water supplemented with 212 mM sucrose and glucose respectively for the inner and outer solutions. Briefly, bacteria were grown in pure LB, harvested after 3.5 h of growth at 37 °C, washed twice by centrifugation (6500 g, 2 min) and diluted in inner solution to OD=0.3. Encapsulation in GUVs followed the protocol detailed in the main text, except that the column was centrifuged at only $400g$ because of the larger density difference between the inner and outer media in these conditions. The imaging chamber was sealed at $\alpha = 1.1$. We saw tubes similar to those observed in the main experiments, and the same coupling between tubes and flagella bundles leading to GUV motion (Fig. S7 (d)-(h)). We conclude that the peptides contained in LB do not play a prominent role in tube formation.

Note that different sugar solutions across the membrane can generate spontaneous curvature. The sucrose and glucose concentrations used here match those used in (7), which generated a spontaneous curvature $m = 1.3\,\mu m^{-1}$. In the presence of spontaneous curvature, the force needed to pull a membrane tube becomes $f = 2\pi\sqrt{2\kappa\sigma} - 4\pi\kappa m$ and the equilibrium tube radius is $R_t \sim \sqrt{\frac{\kappa}{2(\sigma+2\kappa m^2)}}$. Using values from the main text, we find a theoretical minimum radius $R_{t,min} \sim 0.3\,\mu m$, *i.e.*, thinner than the bacteria and in agreement with the pictures displayed in Fig. S7 (d)-(h). However, further control experiments detailed in the next paragraph show that a sugar-induced positive spontaneous curvature is not needed to obtain thin tubes.

Defined buffers, symmetric conditions. As a final control, we repeated the experiment with defined, symmetric conditions. GUVs produced with the protocol described in the previous paragraph were diluted 10 times in deionized water supplemented with 212 mM sucrose. The outer solution was thus almost identical to the inner medium (deionized water supplemented with 212 mM sucrose). Once again, bacteria were observed in thin membrane tubes (Fig. S7 (i) & (j)), indicating that spontaneous curvature is not needed to explain the formation of these tubes.

In sum, these control experiments indicate that a spontaneous curvature of molecular origin (peptides and/or compositional asymmetry across the membrane) is unlikely to be the main factor explaining the spontaneous formation of thin empty tubes or the thinner than expected diameter of bacteria-containing tubes. We cannot fully exclude a potential effect of leftover oil or defects in the membrane but, as explained

in the main text, an initial area difference between the inner and outer leaflets of the membrane could be the main factor aiding tube formation in our system.

Table S1. Analysis of GUV populations at different osmotic deflation factors α.

α	1	1.05	1.1
n	93	134	114
χ (%)	17	49	67
$R_{\mathrm{av}} \pm$ SD	10.8 ± 4.5	10.3 ± 5.4	9.6 ± 3.8
R_{med}	10	9.1	9.5
$[R_{\mathrm{min}}\ ;\ R_{\mathrm{max}}]$ (µm)	[4.3 ; 28.3]	[2.9 ; 34.5]	[3.4 ; 23.9]

n is the total number of GUVs analysed for each α. χ is the fraction of GUVs displaying one or several bacteria-containing tube(s). R_{av}, R_{med}, R_{min} and R_{max} are respectively the number averaged, median, minimum and maximum radii recorded for each population. SD: standard deviation.

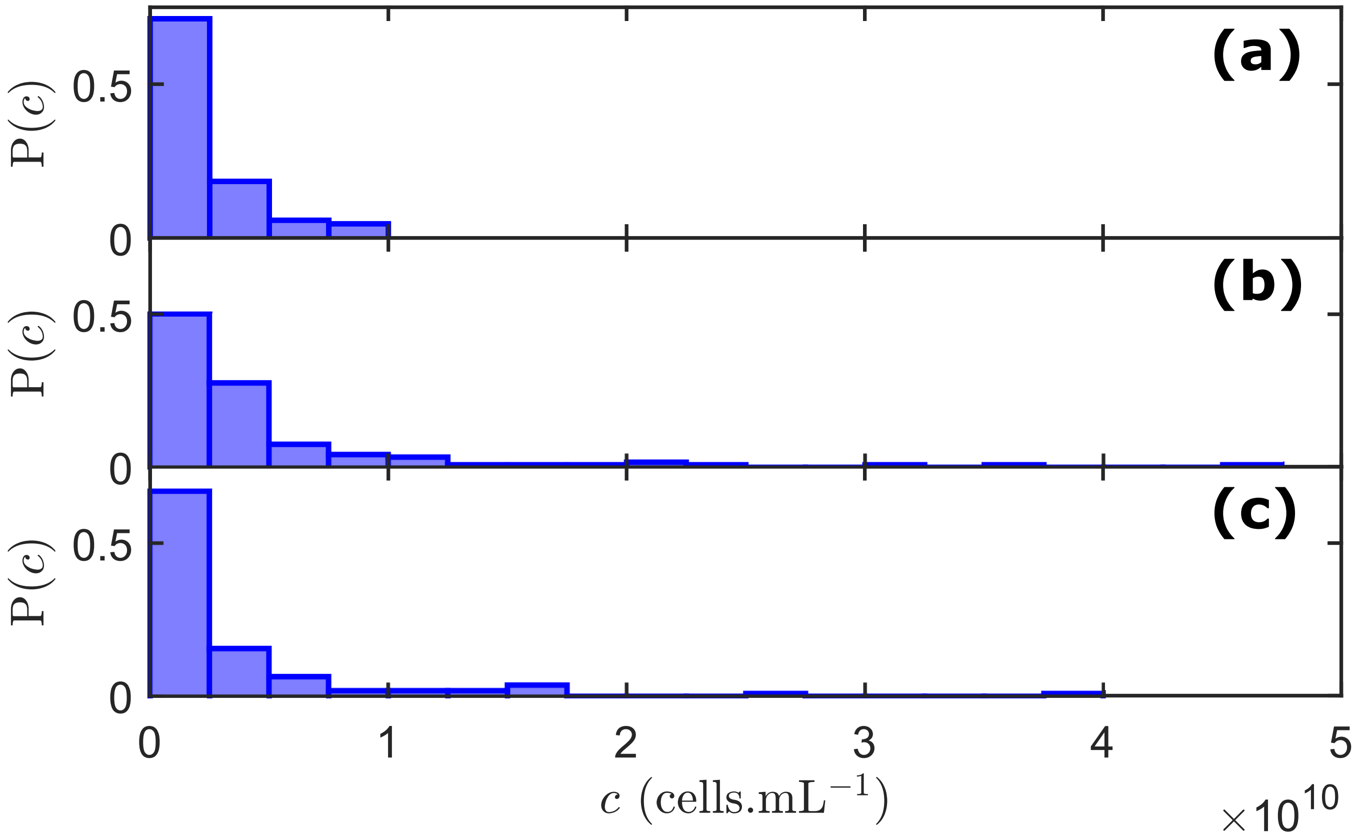

Fig. S1. Probability distributions of cell concentrations measured on the GUV populations studied in Table S1. **(a)** $\alpha = 1$, median concentration $c_{\mathrm{m}} = 1.4 \times 10^9$ cells mL^{-1}. **(b)** $\alpha = 1.05$, median concentration $c_{\mathrm{m}} = 2.6 \times 10^9$ cells mL^{-1}. **(c)** $\alpha = 1.1$, median concentration $c_{\mathrm{m}} = 1.4 \times 10^9$ cells mL^{-1}.

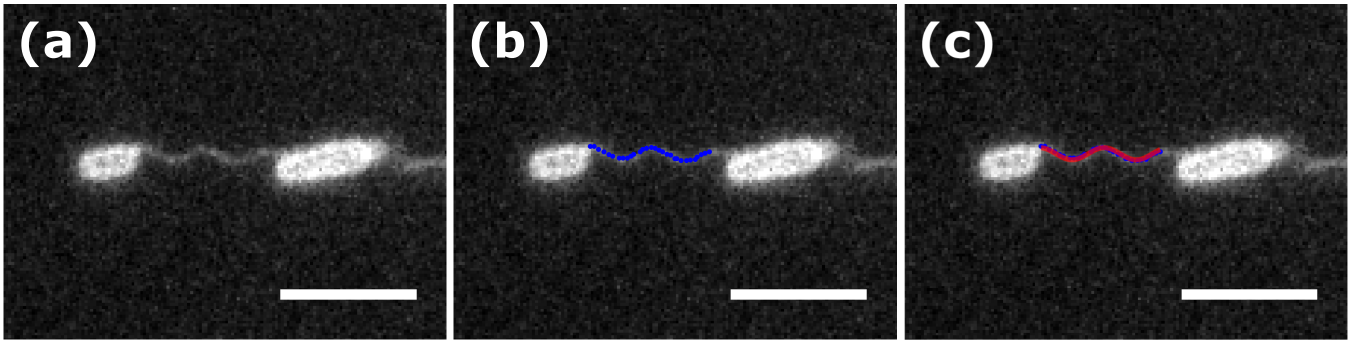

Fig. S2. Image analysis of the flagella bundle. **(a)** Raw image showing a helical, horizontally oriented portion of tube at the back of a small cell, immediately followed by another larger cell. **(b)** Manually extracted tube profile (blue points). **(c)** Fit of the tube profile with a sinusoidal function (superimposed red curve). The function used is $y = \frac{d}{2} \times sin\left(\frac{2\pi x}{p} + c_1\right) + c_2$ and directly returns the diameter $d = 0.44\,\mu\text{m}$ and pitch $p = 2.36\,\mu\text{m}$ of the helical bundle. Bars: $5\,\mu\text{m}$.

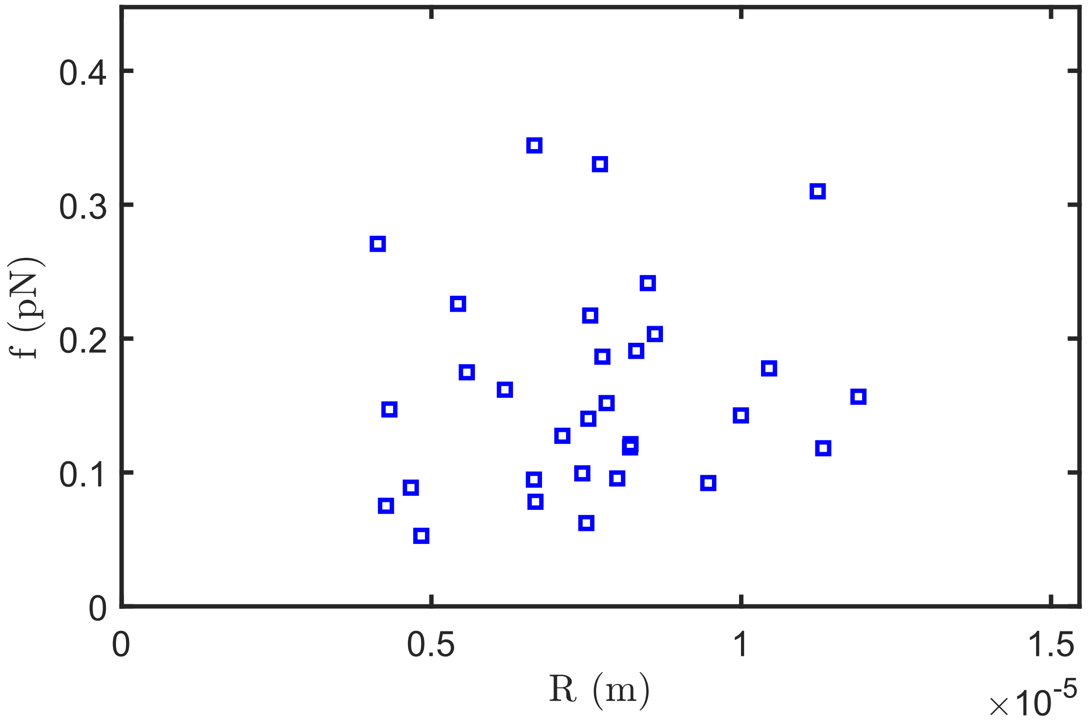

Fig. S3. Calculated propulsive force plotted as a function of GUV radius for all GUVs propelled by a single bacterium. As expected, no correlation is observed between the two variables.

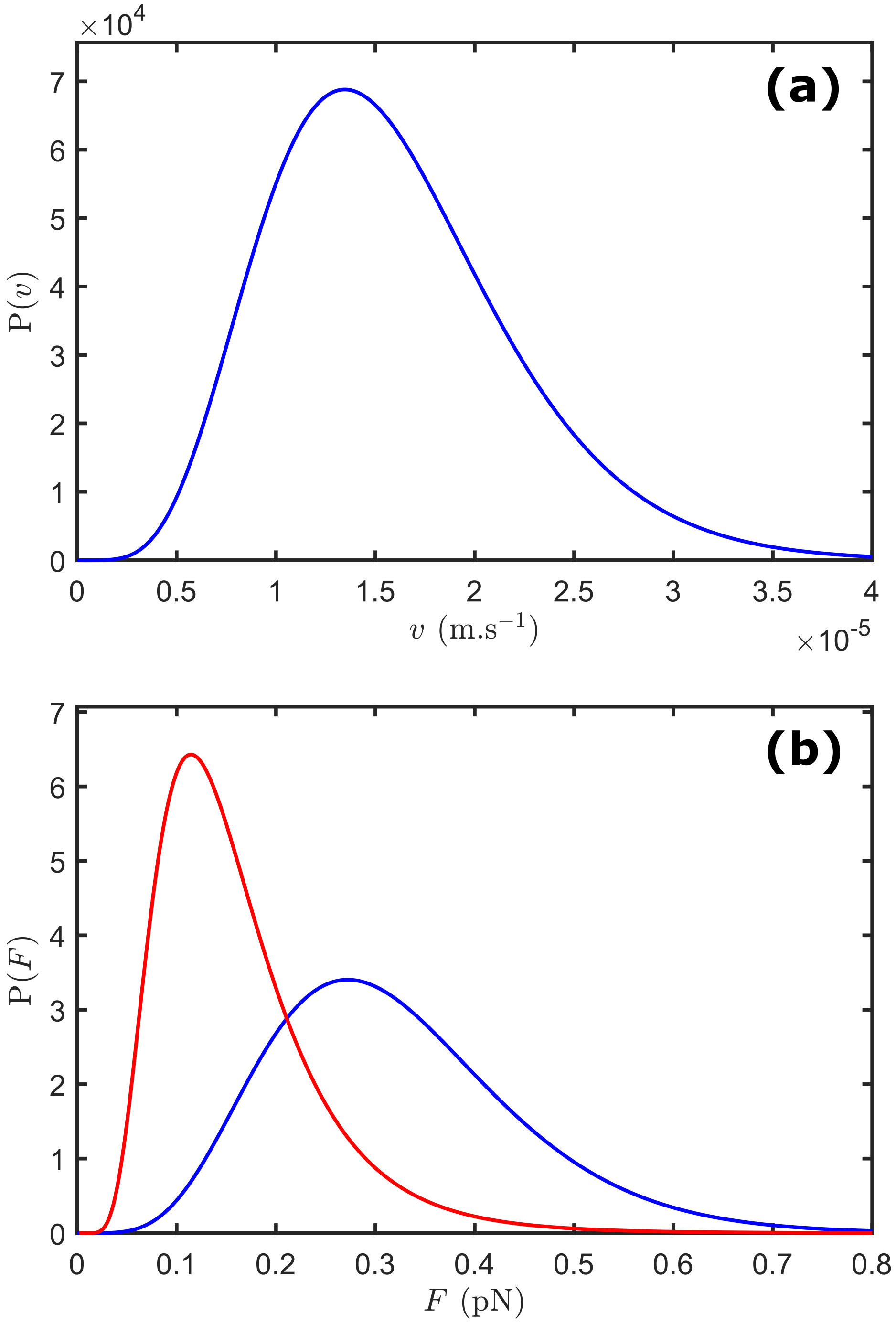

Fig. S4. DDM analysis of freely-swimming cells. **(a)** Schulz distribution of swimming speeds obtained for a suspension of freely-swimming *E. coli* in LB-sucrose, with $\bar{v} = 15.9\,\mu\mathrm{m\,s^{-1}}$ and $\sigma_v = 6.2\,\mu\mathrm{m\,s^{-1}}$. **(b)** Comparison between the thrust force distribution obtained for freely-swimming bacteria (blue line, derived from the speed distribution with $\bar{F} = 0.32\,\mathrm{pN}$ and $\sigma_F = 0.13\,\mathrm{pN}$) and for bacteria in membrane tubes (red line, corresponding to the log-normal distribution of Fig. 4 (a) in the main text). Both distributions have similar coefficients of variation: $c_v = 0.51$ for bacteria in membrane tubes compared to $c_v = 0.39$ for freely-swimming cells.

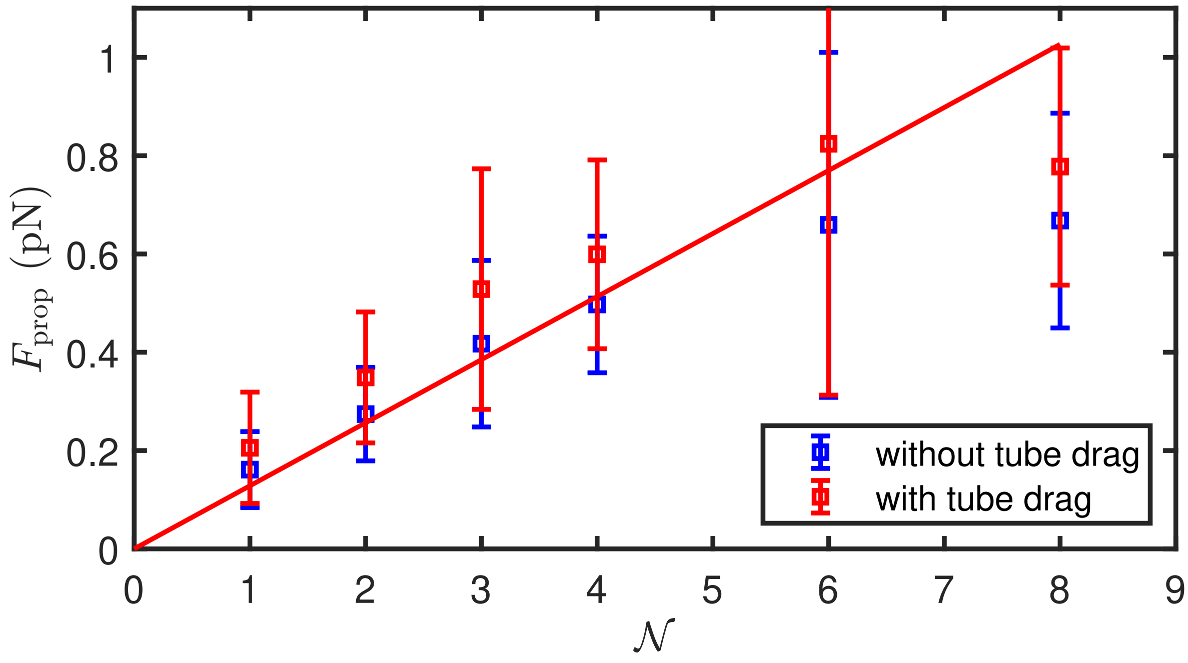

Fig. S5. Average value of the total propulsive force $F_{\rm prop}$ generated by bacteria as a function of the number of bacteria $\mathcal{N}$ in the tubes, with and without taking into account tube drag. Red points are calculated using an upper bound for the drag on the tube, $D_t = 0.4\,\mu\mathrm{m}$. The red line is a linear fit of the red points weighted by the inverse of the variance of the data, with a fitted slope $\langle f \rangle = 0.13\mathcal{N}$ pN ($R^2 = 0.7$). Error bars correspond to standard deviations.

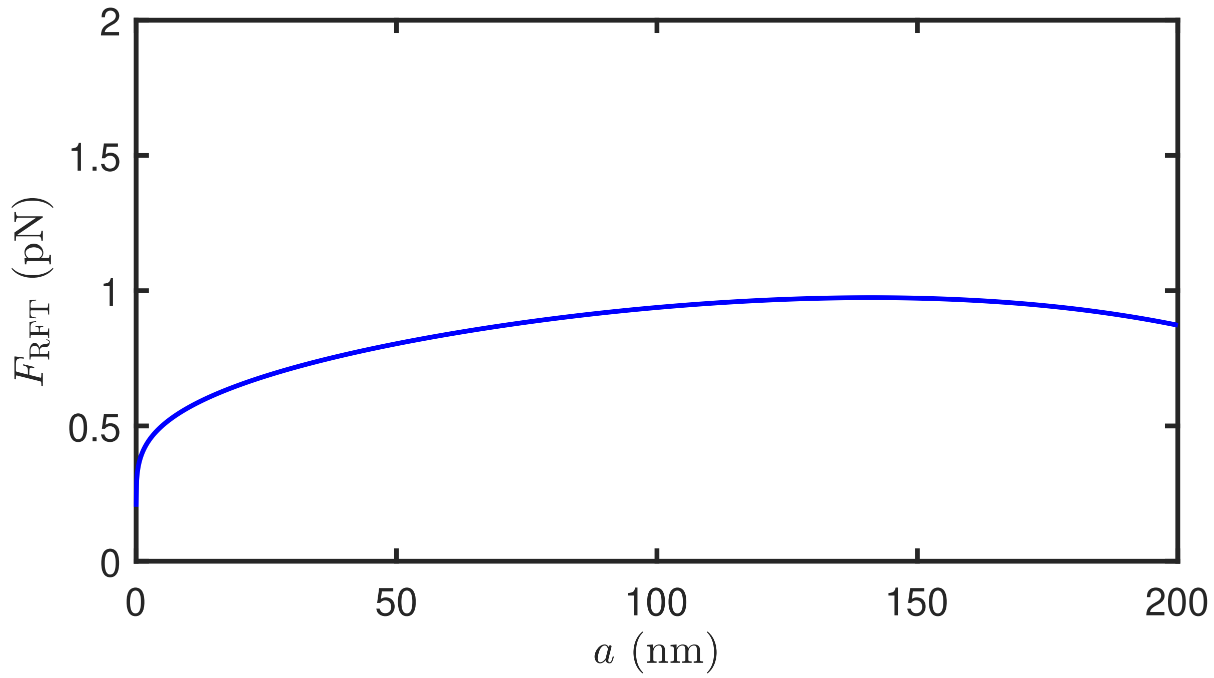

Fig. S6. The thrust force F_{RFT} calculated for a helix of thickness $2a$ using resistive force theory varies weakly with a over the plausible range covered in experiments, $25\,\mathrm{nm} < a < 200\,\mathrm{nm}$.

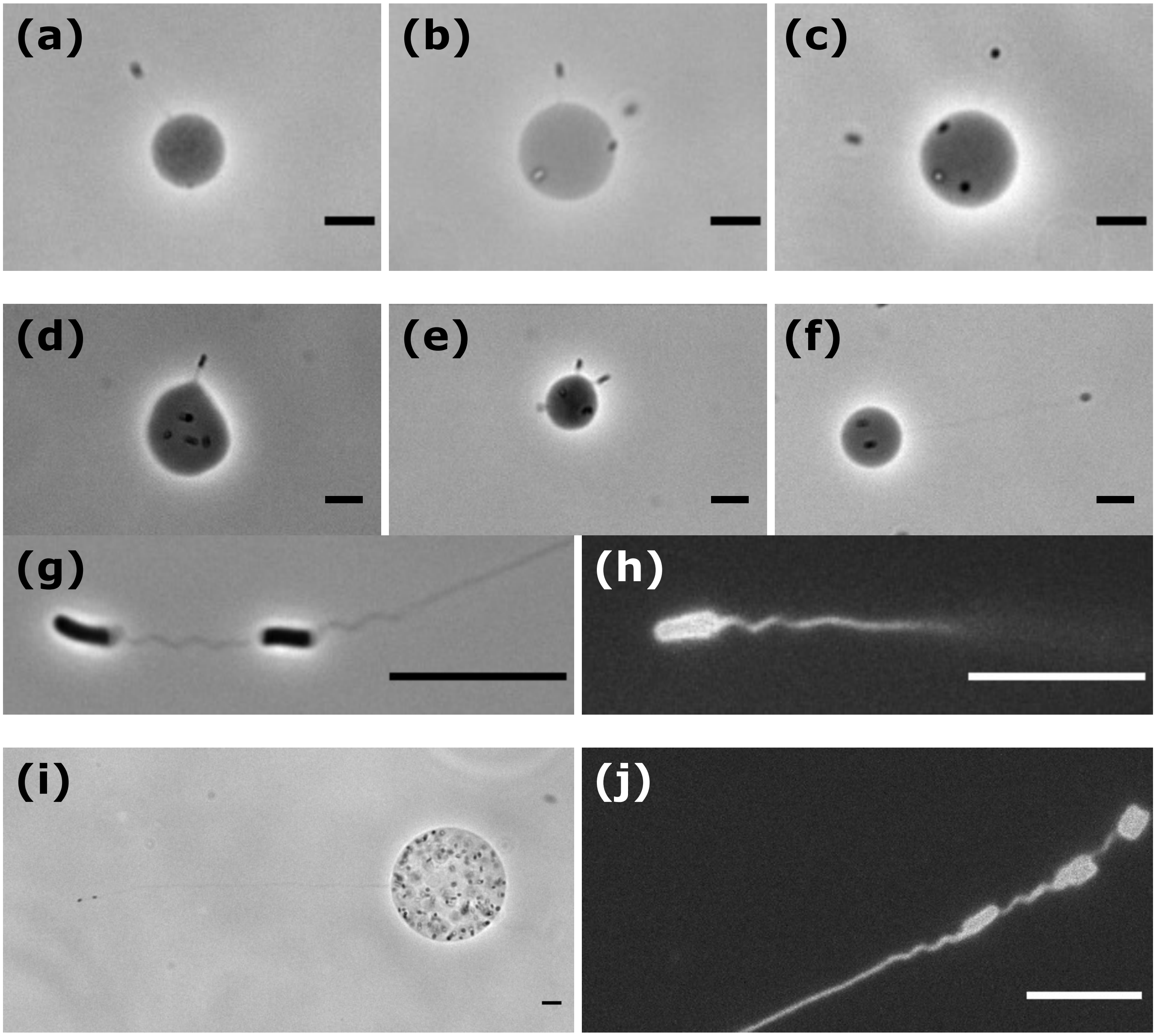

Fig. S7. Thin tubes coupling with flagella obtained in three independent experiments using different IS and OS (see text for details). **(a)-(c)** Symmetric conditions using sucrose-supplemented LB. **(d)-(h)** Asymmetric conditions using an IS made of sucrose supplemented deionized water and an OS made of glucose supplemented deionized water. **(i),(j)** Symmetric conditions using sucrose-supplemented deionized water. Bars: $10\,\mu\mathrm{m}$.

Movie S1. Swimming vesicles propelled by bacteria in membrane tubes. Accelerated 10x. Bar: 20 µm.

Movie S2. Fast rotation of a helical membrane tube with the encapsulated flagella bundle. Slowed down 40x. Bar: 10 µm.

Movie S3. Thicker tube of Fig. 5(a) coupling with the enclosed flagella bundle and undergoing helical motion. Slowed down 40x. Bar: 5 µm.

Movie S4. Split tube with sliding junction point as cells rotate their flagella. Slowed down 10x. Bar: 5 µm.

Movie S5. Cell divisions in a membrane tube. Bar: 20 µm.

References

1. H Cazzola, et al., The impact of plasma membrane lipid composition on flagellum-mediated adhesion of enterohemorrhagic *Escherichia coli*. *MSphere* **5** (2020).
2. VA Martinez, et al., Differential dynamic microscopy: A high-throughput method for characterizing the motility of microorganisms. *Biophys. J.* **103**, 1637–1647 (2012).
3. LG Wilson, et al., Differential dynamic microscopy of bacterial motility. *Phys. Rev. Lett.* **106**, 018101 (2011).
4. R Cox, The motion of long slender bodies in a viscous fluid part 1. general theory. *J. Fluid mechanics* **44**, 791–810 (1970).
5. S Chattopadhyay, R Moldovan, C Yeung, X Wu, Swimming efficiency of bacterium *Escherichia coli*. *Proc. Natl. Acad. Sci.* **103**, 13712–13717 (2006).
6. B Rodenborn, CH Chen, HL Swinney, B Liu, H Zhang, Propulsion of microorganisms by a helical flagellum. *Proc. Natl. Acad. Sci.* **110**, E338–E347 (2013).
7. T Bhatia, S Christ, J Steinkühler, R Dimova, R Lipowsky, Simple sugars shape giant vesicles into multispheres with many membrane necks. *Soft Matter* **16**, 1246–1258 (2020).
8. M Karimi, et al., Asymmetric ionic conditions generate large membrane curvatures. *Nano Lett.* **18**, 7816–7821 (2018).
